# Virtual Assessment of Coronary Microcirculation Before and After Rotational Atherectomy

**DOI:** 10.14740/cr2276

**Published:** 2026-10-24

**Authors:** Gueladio Kone, Brahim Baala, Alexandre Canville, Matthieu Godin, Quentin Landolff

**Affiliations:** a Department of Interventional Cardiology, Clinique Saint-Hilaire, Rouen, France

**Keywords:** Rotablator, Angiography-derived microvascular resistance, Coronary microvascular dysfunction

## Abstract

**Background:**

Calcified coronary lesions remain a major challenge during percutaneous coronary intervention (PCI). Rotational atherectomy (RA) (Boston Scientific) is a well-established tool used to modify calcified plaques and facilitate stent delivery. Despite its mechanical benefits, RA may adversely affect coronary microcirculation through distal plaque debris embolization. Angiography-derived microvascular resistance (AMR) (Pulse Medical) is a wire-free index correlated with invasive indices such as the index of microcirculatory resistance (IMR), which allows assessment of microcirculatory resistance directly from angiographic images, without a pressure wire or adenosine. The objective of this study is to evaluate changes in AMR before and after RA and to identify clinical, angiographic, and procedural predictors of postprocedural coronary microvascular dysfunction (CMD).

**Methods:**

We performed a retrospective single-center observational study including all patients who underwent RA between January 2023 and December 2024 at Clinique Saint-Hilaire in Rouen, France. AMR was measured before and after RA using the AngioPlus software (Pulse Medical). CMD was defined as postprocedural AMR > 250 mm Hg·s/m.

**Results:**

Of 49 RA procedures, 40 patients were included. AMR increased in all cases but CMD occurred in 42.5% of patients (n=17). Diabetes mellitus and reduced left ventricular ejection fraction (LVEF) were significantly more prevalent in the CMD group. Long coronary artery lesions (> 20 mm), larger plaque burden, and prolonged burr run time were associated with higher AMR values. Mean AMR rose from 176 to 315 mm Hg·s/m in the CMD group, versus 177 to 201 mm Hg·s/m in the non-CMD group.

**Conclusions:**

Performing RA affects the coronary microcirculation, especially in cases of diabetes mellitus and reduced LVEF.

## Introduction

Coronary artery disease (CAD) is a major cause of morbidity and mortality worldwide. Calcified coronary lesions represent around 30% of coronary lesions, particularly prevalent in elderly and diabetic populations. Percutaneous coronary interventions (PCI) on these lesions can be challenging, impairing balloon dilatation, limiting stent expansion, and increasing the risks of stent restenosis and thrombosis.

In this context, rotational atherectomy (RA) (Boston Scientific) represents a well-established lesion preparation tool, modifying heavily calcified plaques, facilitating stent delivery and expansion. However, this technique may release plaque debris that can obstruct the distal microcirculation, causing slow-flow or no-reflow phenomena and potentially reducing left ventricular function.

The assessment of coronary microcirculation traditionally relied on invasive wire-based indices such as coronary flow reserve (CFR) or the index of microcirculatory resistance (IMR) measured by Coroflow software (Abbott), which require a pressure wire and pharmacologically induced hyperemia. An IMR value above 25 is accepted for microvascular dysfunction. Although validated, these methods still are underused due to their logistic complexity and cost. Recently, angiography-derived microvascular resistance (AMR) measured by AngioPlus software (Pulse Medical) emerged as a wire-free, rapid, and reproducible alternative that correlates well with IMR (Fig. 1), with a postprocedural AMR ≥ 250 mm Hg·s/m being associated with microvascular dysfunction.

**Figure 1:**
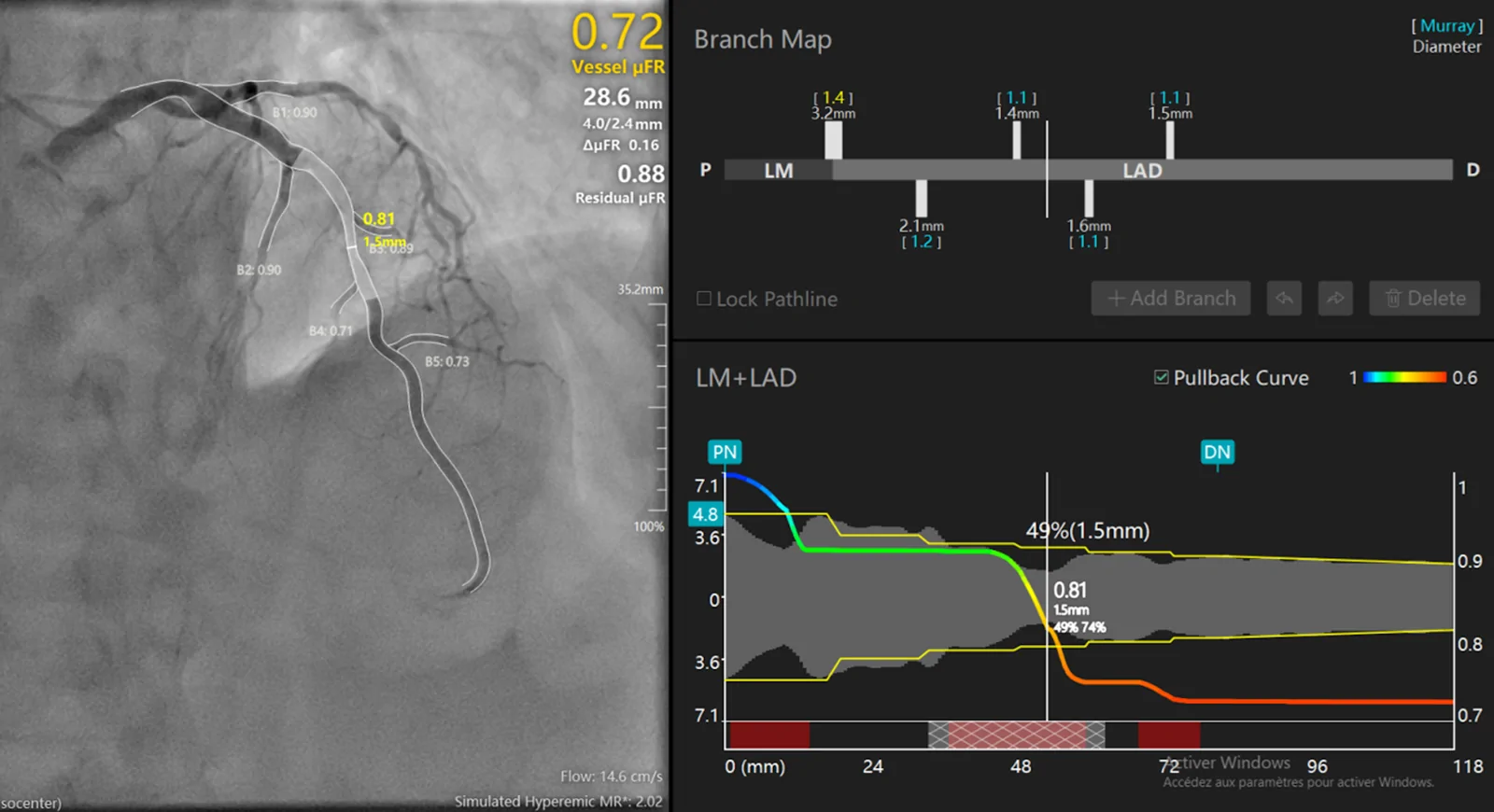
Example of microFR and AMR measurements with AngioPlus (Pulse Medical). Left panel: angiogram with the reconstructed LM–LAD. B1 to B5 designate the side branches identified along the vessel, with the microFR value computed at the level of each branch. The yellow value (0.81; 1.5 mm) indicates the microFR and the minimal lumen diameter at the site of the lesion. Upper-right values: vessel microFR (0.72), lesion length (28.6 mm), proximal/distal reference diameters (4.0/2.4 mm), pressure drop across the lesion = 0.16 (it is the variation of microFR after treatment ΔmicroFR) and predicted residual microFR after treatment of the lesion (0.88). Lower values: estimated contrast flow velocity (14.6 cm/s) and simulated hyperemic microvascular resistance, AMR (2.02 mm Hg·s/cm, corresponding to 202 mm Hg·s/m). Right upper panel (branch map): schematic representation of the LM and LAD from proximal (P) to distal (D), showing the position and diameter of each side branch. Right lower panel (LM + LAD pullback curve): virtual microFR pullback along the vessel. The vertical line marks the minimal lumen diameter site (1.5 mm; 49% diameter stenosis and 74% area stenosis; microFR, 0.81). AMR: angiography-derived microvascular resistance; LAD: left anterior descending artery; LM: left main coronary artery; microFR: Murray-law-based quantitative flow ratio.

The aim of this study was to evaluate the impact of RA on coronary microcirculation using AMR and to identify clinical, angiographic, and procedural factors associated with postprocedural microvascular dysfunction. We hypothesized that RA induces a measurable increase in AMR, reflecting coronary microvascular injury.

## Materials and Methods

### Study design and population selection

This retrospective, single-center, non-randomized study was conducted at the Department of Interventional Cardiology, Clinique Saint-Hilaire, Rouen, France from January 2023 to December 2024. In this period, 49 RA procedures were performed. Inclusion criteria were age > 18 years; at least one severely calcified coronary lesion treated with RA; angiographic image quality sufficient for AMR analysis (acquisition rate ≥ 7.5 frames/s) both before and after the procedure, and availability of 1-year clinical follow-up through the France PCI registry. Exclusion criteria included prior bypass grafting of the target vessel, poor angiographic image quality, and ST-elevation myocardial infarction (STEMI) (Fig. 2).

**Figure 2:**
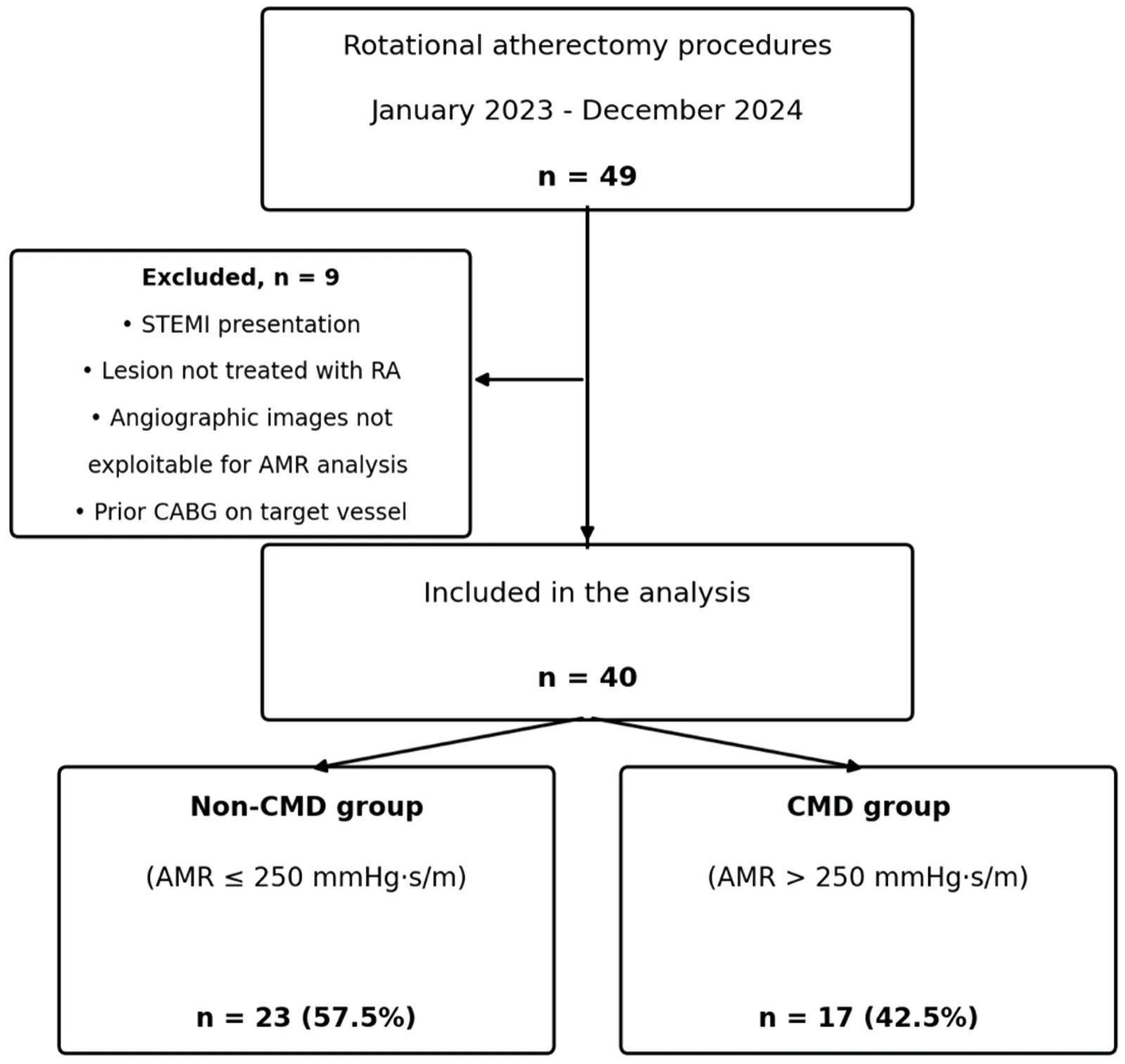
Flow chart of the study. AMR: angiography-derived microvascular resistance; CABG: coronary artery bypass grafting; CMD: coronary microvascular dysfunction; RA: rotational atherectomy; STEMI: ST-elevation myocardial infarction.

We applied these exclusion criteria because of factors that could compromise the validity of AMR analysis, such as distal coronary flow disturbance in patients with coronary artery bypass grafting (CABG), suboptimal angiographic image quality (non-Thrombolysis in Myocardial Infarction (TIMI) 3 flow, tortuosity), and transient microvascular injury in patients with STEMI.

### Procedural protocol

RA was performed with the Rotablator system (Boston Scientific, Marlborough, MA, USA) by an experienced operator, according to standardized departmental practice and current recommendations. After lesion crossing with a dedicated RotaWire, a burr size of 1.25–1.75 mm was selected, targeting a burr-to-artery ratio close to 0.5. Burr rotation was initiated at low speed (60,000–70,000 revolutions per minute (rpm)) before engaging the lesion, then increased to a working speed of 130,000–180,000 rpm (up to ≥ 180,000 rpm when dilatation remained incomplete); each run was limited to less than 30 s. Anticoagulation followed guidelines [1].

### AMR analysis

The analysis was performed with AngioPlus software (Pulse Medical) using a validated algorithm that estimates distal pressure and hyperemic velocity from angiographic flow. AMR was measured before and after RA. Values ≥ 250 mm Hg·s/m after RA were considered abnormal. A threshold based on a validated cutoff was compared with invasively measured coronary microcirculatory resistance indices [2, 3].

AMR was calculated as follows: AMR = P_d_/V_hyp_ = P_a_ × microFR/V_hyp_ [4], where P_d_ is the distal coronary pressure (mm Hg), P_a_ is the mean aortic pressure (mm Hg) recorded at the tip of the guiding catheter; microFR is the Murray-law-based quantitative flow ratio, and V_hyp_ is the hyperemic mean flow velocity (m/s) derived from the contrast. AMR is therefore expressed in mm Hg·s/m.

Because AMR relies on accurate three-dimensional vessel reconstruction, its reliability can be affected by suboptimal postprocedural image quality (e.g., contrast staining, residual dissection flap, or transiently reduced flow). All angiograms included in the analysis, before and after RA, met the same quality criteria; studies not meeting this standard were excluded. We acknowledge that a dedicated technical validation of AngioPlus specifically after RA has not been performed to date and remains needed.

### Data collection

Clinical, angiographic, and procedural data were collected retrospectively. Statistical analyses were performed using Microsoft Excel and ODK Central. ODK Central is a platform used as a standardized and secure electronic data-capture server.

### Statistical analysis

Continuous variables are expressed as mean ± standard deviation and were compared between the coronary microvascular dysfunction (CMD) and non-CMD groups using Student’s *t*-test. The paired difference between preprocedural and postprocedural AMR (ΔAMR) was assessed using a paired *t*-test

### Follow-up

One-year clinical follow-up data were collected from the France PCI registry.

### Ethics

The Institutional Review Board (IRB) approved the study. This study was conducted in compliance with the ethical standards of the responsible institution on human subjects as well as with the Helsinki Declaration.

## Results

### Population characteristics

Mean age was 75 years, and 87% were male. Among the cardiac risk factors, diabetes mellitus was present in 47% overall and was more prevalent in in the CMD group (70% vs 30%).

Reduced left ventricular ejection fraction (LVEF) was more common in the CMD group (41% vs 17%). Other cardiovascular risk factors, comorbidities, and clinical presentation were comparable between groups (Table 1).

**Table 1:** Clinical Characteristics

Variable	Non-CMD group (n = 23)	CMD group (n = 17)	P
Age, years (mean ± SD)	76.1 ± 9	74.1 ± 9	0.501
Male sex, n (%)	19 (83)	16 (94)	0.37
Diabetes mellitus, n (%)	7 (30)	12 (70)	0.028
Hypertension, n (%)	19 (82)	14 (82)	1
Dyslipidemia, n (%)	10 (40)	10 (58)	0.52
Current/former smoking, n (%)	13 (56)	11 (64)	0.74
Chronic kidney disease, n (%)	1 (4)	2 (11)	0.56
Prior myocardial infarction, n (%)	5 (21)	3 (17)	1
Prior PCI, n (%)	7 (30)	8 (47)	0.45
Prior CABG, n (%)	1 (4)	0 (0)	1
Atrial fibrillation, n (%)	2 (8)	3 (17)	0.63
Prior stroke, n (%)	4 (17)	4 (23)	0.70
Stable angina, n (%)	13 (57)	13 (77)	0.21
Acute coronary syndrome, n (%)	10 (43)	4 (23)	1
Preserved LVEF (≥ 50%), n (%)	19 (83)	10 (59)	0.15
Pre-procedural AMR, mm Hg·s/m (mean)	177	176	—
Postprocedural AMR, mm Hg·s/m (mean)	201	315	—

AMR: angiography-derived microvascular resistance; CABG: coronary artery bypass grafting; CMD: coronary microvascular dysfunction; LVEF: left ventricular ejection fraction; PCI: percutaneous coronary interventions; SD: standard deviation.

### Procedural findings

Radial access was used in 97% of cases.

Target coronary artery lesions were located in the left anterior descending artery (LAD) in 65% of cases, the right coronary artery (RCA) in 27%, and the left circumflex artery (LCX) in 8%. Left main coronary artery lesions were present in 10% of the overall population, without significant difference between the two groups.

Lesions longer than 20 mm were more prevalent in the CMD group (70%). Multivessel disease was frequent in both groups.

A 1.5-mm burr was used in the majority of cases and comparably in both groups. The 1.75-mm burr was used exclusively in the CMD group (two patients) (Table 2).

**Table 2:** Procedural Characteristics

Variable	Non-CMD group (n = 23)	CMD group (n = 17)	Between-group comparison
Radial access, n (%)	39/40 (97.5) overall		NS^a^
Target lesion, n (%)			
LAD	26/40 (65) overall		NS^a^
RCA	11/40 (27) overall		NS^a^
LCX	3/40 (8) overall		NS^a^
Lesion length > 20 mm, n (%)	13 (56)	12 (70)	Not formally tested^b^
Three-vessel disease, n (%)	Majority in both groups		NS^a^
Left main lesion, n (%)	4/40 (10) overall		NS^a^
Intracoronary imaging (OFDI), n (%)	7/40 (17.5) overall		NS^a^
Burr size 1.5 mm, n (%)	17 (74)	12 (70)	Not formally tested^b^
Burr size 1.75 mm, n (%)	0 (0)	3 (18)	Not formally tested^b^
Mean number of RA passes	2	4	Not formally tested^b^
Burr run time, s	20 (median)	60 (mean)	Not formally tested^b^

^a^NS: not significant (P ≥ 0.05) for the comparison between the non-CMD and CMD groups; for these variables, values are reported for the overall population (n = 40). ^b^Not formally tested: no statistical test was performed for this variable because of the small number of patients per group; values are presented descriptively. LAD: left anterior descending artery; CMD: coronary microvascular dysfunction; LCX: left circumflex artery; OFDI: optical frequency-domain imaging; RA: rotational atherectomy; RCA: right coronary artery.

### Microcirculation result findings

AMR increased in all patients after RA (100%), rising from 177 to 201 mm Hg·s/m in the non-CMD group and from 176 to 315 mm Hg·s/m in the CMD group. This result suggests that AMR may serve as a marker of coronary microvascular injury (Table 1).

### Complications

Coronary artery dissections occurred only in the CMD group (17%).

Follow-up data were available for all 40 patients through the France PCI registry. During the 1-year follow-up, three long-term events were recorded from the France PCI registry, all in the non-dysfunction group: one death, one myocardial infarction, and one stroke.

## Discussion

This study demonstrates that RA is consistently associated with a rise in coronary microvascular resistance with significant variation according to patient and lesion characteristics and with CMD occurring in 42.5% of patients.

Patients who developed CMD after RA more frequently presented with cardiovascular risk factors such as diabetes mellitus and current smoking. This is consistent with the results of Williams et al, who demonstrated that chronic hyperglycemia and high glycated hemoglobin levels are known to impair endothelial function and red cell rheology, both of which could plausibly contribute to increased resistance in the coronary microcirculation [5]. In our study, diabetes mellitus was found to be an important predictive factor for CMD. Other classical risk factors, such as hypertension and dyslipidemia, were numerically more frequent in the CMD group but did not reach statistical significance.

Procedural characteristics, particularly the length of the coronary artery lesion treated with RA, were identified as major determinants of CMD in our study. It often reflects the presence of more severe calcification and a larger plaque burden, which are responsible for an increased production of debris during RA.

Wang et al reported CMD in 45.8% of cases after RA, particularly among patients with long or multivessel coronary artery lesions [6]. In our study, 70% of patients with CMD after RA had coronary artery lesions longer than 20 mm. This comparison should be interpreted cautiously, as the two cohorts differ in sample size and in the microcirculatory index used: Wang et al [6] assessed an angiography-derived IMR rather than AMR.

Several techniques are available to assess coronary microvascular resistance. Invasive approaches such as IMR remain the reference due to their diagnostic accuracy, particularly when the IMR value exceeds 25 [7]. However, IMR presents certain limitations, including the use of hyperemic agents and pressure wires, which lengthen the procedure and increase its cost. Likewise, cardiac magnetic resonance imaging (MRI) is considered the gold standard for evaluating myocardial perfusion and tissue viability. However, it faces logistical constraints, including limited availability, long examination times, and high costs.

To overcome these limitations, AMR has emerged as a promising alternative. AMR is a virtual software-based tool that requires neither a pressure wire nor adenosine, and it is derived from angiography, allowing a simplified assessment of coronary microvascular resistance. This method offers a more accessible and cost-effective solution [8]. Prior validation work, including studies in patients with chronic coronary syndromes [3] and studies using computational fluid-dynamics models comparable to those underlying AMR [4, 9], has reported good correlations between angiography-derived and invasively measured microcirculatory indices. Nonetheless, dedicated validation of AMR specifically in the post-RA setting is still lacking and represents a priority for future research.

In our study, post–RA AMR was significantly higher in patients with CMD, with mean AMR increasing from 176 to 315 mm Hg·s/m in the CMD group.

### Clinical implications

Low-flow and no-reflow during RA are recognized complications secondary to microvascular injury. Strategies to minimize coronary microvascular injury may include minimizing rotational time, using smaller burr sizes, and giving adjunctive vasodilators.

The study is limited by its retrospective design, modest sample size, and lack of direct IMR comparison.

AMR accuracy also depends on angiographic quality.

### Limitations

This study has several limitations. It is retrospective, single-center, and non-randomized; the sample size is modest. No direct invasive IMR comparison was available. Although AMR has shown good correlation with IMR in prior validation studies, dedicated technical validation of AngioPlus specifically in the post-RA setting has not been performed. No biological markers of myocardial injury (e.g., troponin) were collected. Given the very small number of events, no formal statistical outcome analysis was performed; we chose to retain these events as a descriptive, hypothesis-generating observation. No comparator group (balloon angioplasty or another calcified-plaque-modification technique) was included. Finally, AMR calculation quality depends on angiographic acquisition quality, which may itself be affected after RA.

### Conclusions

RA systematically increases AMR, reflecting coronary artery microvascular stress, with coronary artery dysfunction in nearly half of patients. Diabetes mellitus, reduced LVEF, coronary artery lesion length and prolonged RA duration are key predictors of CMD. AMR is a reliable, wire-free tool for evaluating coronary microcirculation and may enhance decision-making during complex PCI.

Future multicenter prospective trials should compare AMR with IMR and assess long-term prognostic value. Integration of AMR in PCI workflows will allow better patient selection and improve outcomes in complex calcified CAD.

## Data Availability

The data supporting the findings of this study are available from the corresponding author upon reasonable request.

## Abbreviations

AMR: angiography-derived microvascular resistance
CAD: coronary artery disease
CFR: coronary flow reserve
IMR: index of microcirculatory resistance
LAD: left anterior descending artery
LCX: left circumflex artery
LVEF: left ventricular ejection fraction
MRI: magnetic resonance imaging
microFR: Murray-law-based quantitative flow ratio
PCI: percutaneous coronary interventions
RA: rotational atherectomy
RCA: right coronary artery
